# Splenic Macrophage Subsets and Their Function during Blood-Borne Infections

**DOI:** 10.3389/fimmu.2015.00480

**Published:** 2015-09-22

**Authors:** Henrique Borges da Silva, Raíssa Fonseca, Rosana Moreira Pereira, Alexandra dos Anjos Cassado, José Maria Álvarez, Maria Regina D’Império Lima

**Affiliations:** ^1^Department of Immunology, Instituto de Ciências Biomédicas, Universidade de São Paulo, São Paulo, Brazil

**Keywords:** spleen, macrophages, phagocytosis, pattern-recognition receptors, tissue remodeling

## Abstract

The spleen is one of the major immunological sites for maintaining blood homeostasis. Previous studies showed that heterogeneous splenic macrophage populations contribute in complimentary ways to control blood-borne infections and induce effective immune responses. Marginal metallophilic macrophages (MMMΦs) and marginal zone macrophages (MZMΦs) are cells with great ability to internalize blood-borne pathogens such as virus or bacteria. Their localization adjacent to T- and B-cell-rich splenic areas favors the rapid contact between these macrophages and cells from adaptive immunity. Indeed, MMMΦs and MZMΦs are considered important bridges between innate and adaptive immunity. Although red pulp macrophages (RpMΦs) are mainly considered scavengers for senescent erythrocytes, several data indicate a role for RpMΦs in control of infections such as blood-stage malaria as well as in the induction of innate and adaptive immunity. Here, we review current data on how different macrophage subsets recognize and help eliminate blood-borne pathogens, and, in turn, how the inflammatory microenvironment in different phases of infection (acute, chronic, and after pathogen clearance) influences macrophage function and survival.

## Introduction

**Figure 1:**
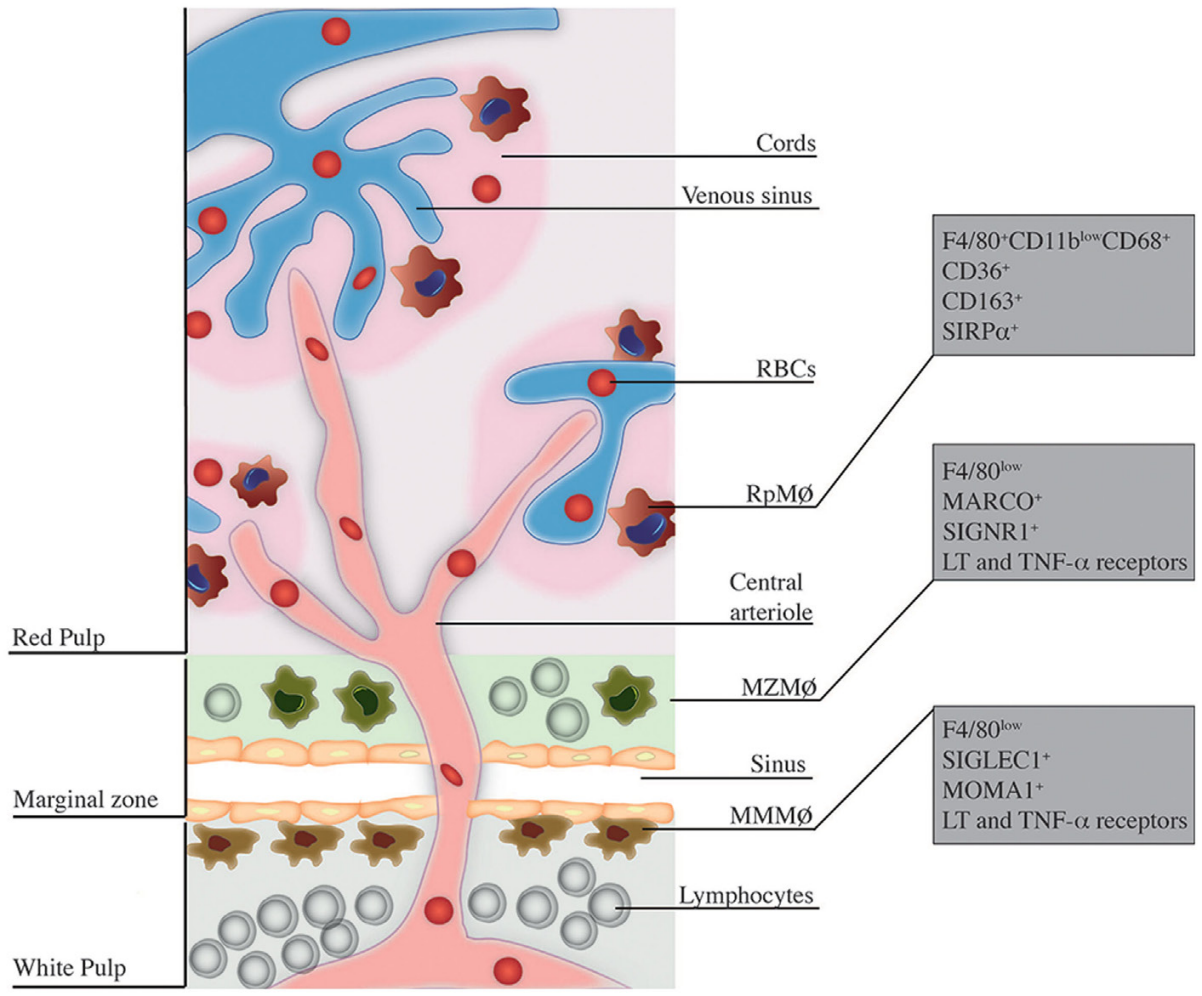
**Localization and phenotype of splenic MΦ subsets**. This figure is a broad scheme of the positioning of RpMΦs, MZMΦs, and MMMΦs inside spleen and their respective phenotypic markers. RpMΦs (in red) are typically found within cords on the red pulp, allowing direct contact with RBCs and other blood cells/particles passing through venous sinuses. They are better defined by the concomitant expression of F4/80, CD11b (at low levels), and CD68 as well as other receptors that aid in their function. MZMΦs (in green) are found in the marginal zone (MZ) outer layer – they are also in direct contact with blood-borne particles. These cells express in their surface the molecules MARCO and SIGNR1 and other receptors that help in the uptake of blood-borne pathogens. Finally, the MMMΦs (in brown) reside within the inner layer of the MZ, in the contact with the white pulp. They are also specialized in blood-borne particle uptake and express surface markers such as SIGLEC-1 and MOMA-1.

Effective control of infections through the immune system is ensured by the well-organized ­structure of secondary lymphoid organs, which allow capture, processing, and presentation of antigens, ultimately leading to successful elimination of pathogens and induction of adaptive immunity. Among lymphoid organs, the spleen is particularly shaped for clearance of blood-borne pathogens. Microanatomically, the spleen is divided into the white pulp and the red pulp (Rp), separated by the marginal zone (MZ) [reviewed in Ref. (1)]. Rp and MZ have a complex macrophage (MΦ) network with distinct origins and functions in the immune response to infections. RpMΦs form a vast network inside the Rp and are characterized in mice by expression of F4/80^high^CD68^+^CD11b^low/−^ and intense autofluorescence (2). In turn, inside the MZ, two populations of MΦs can be discerned. The MZMΦs typically express in their surface the C-type lectin SIGN-related 1 (SIGNR1) and a type I scavenger receptor called Macrophage Receptor with Collagenous structure (MARCO), which recognize non-opsonized molecules (3), mainly blood-borne antigens (4). Furthermore, marginal metallophilic MΦs (MMMΦs) are defined, among other molecules, by the expression of Sialic acid-binding Ig-like Lectin-1 (Siglec-1, Sialoadhesin, CD169) and MOMA-1 (5). A general scheme of the spleen structure is depicted in Figure 1.

Recent studies led to a growing understanding of the precise roles different splenic MΦs play to maintain blood homeostasis, particularly in infectious diseases, in which pathogen elimination depends on the development of appropriate adaptive immune response. In this review, we addressed the roles of each one of these MΦ subsets, with special focus on blood-borne infections. We described the current knowledge on the effects of splenic microarchitecture and microenvironment on these MΦs and reciprocal influence of these cells on spleen structure and functionality.

## How Splenic MΦ Sense Pathogens and Damage-Associated Self-Molecules?

Splenic MΦs have two main protective activities during blood-borne infections. The first and most well characterized is phagocytosis and elimination of pathogens from circulation. However, beyond the task of eliminating blood-borne pathogens, splenic MΦs can play a prominent role in immune system activation. To properly execute these functions, they are provided with a large variety of pattern-recognition receptors (PRRs) that recognize pathogen-associated molecular patterns (PAMPs) and damage-associated molecular patterns (DAMPs). Engagement of Toll-like receptor (TLR) 4 by pathogen molecules, such as lipopolysaccharides (LPS) from Gram-negative bacteria is fundamental for the induction of a proinflammatory gene and protein expression signature in MΦs, which ultimately leads to innate immune activation (6). This also holds true for several other interactions such as TLR2 and/or TLR4 with glycosylphosphatidylinositol (GPI) anchors from *Trypanosoma* and *Plasmodium* parasites (7, 8) and TLR9 engagement by CpG motifs found in bacterial (9) and plasmodial DNA (10).

On the other hand, TLRs recognize DAMPs in situations of tissue injury. For example, heat shock proteins (HSPs) are endogenous damage signals (molecules released by cells under stress or necrotic cell death) and bind to TLR2 and TLR4 in MΦs, inducing these cells to produce proinflammatory cytokines and to express costimulatory molecules (11). Release of HSPs to circulation has been reported during sepsis (12) as well as production of HSP homologues by pathogens such as *Plasmodium* parasites (13). Also, TLRs – especially TLR2 and TLR4 – can recognize extracellular matrix components such as fibronectin (14). TLR4 engagement by fibronectin leads to MΦ activation in a similar fashion to what happens after LPS stimulation. Fibronectin is presumably secreted by fibroblasts inside the spleen. Thus, this molecule may be produced during blood-borne infections such as malaria, where profound changes in splenic microarchitecture following acute infection occur, leading to the accumulation of fibroblasts inside the Rp (15). Expression of fibronectin-binding proteins (FnBPs) by *Staphylococcus aureus* is important to bacterial uptake by MΦs in inflammatory situations through binding of very late antigen 5 (VLA-5) (16). Therefore, it is reasonable to question whether TLR2 and/or TLR4 expressed in MΦs are engaged by fibronectin in those situations. Importantly, *S. aureus* FnBPs are crucial for the development of sepsis (16).

Another DAMP that can induce MΦ activation is the high mobility group box protein 1 (HMGB1), an intracellular DNA-binding protein involved in chromatin remodeling and transcription regulation (17). Extracellular HMGB1 binds to different endogenous ligands that are recognized by receptors such as TLR4, as well as the receptor for advanced glycation end products (RAGE) (18), and triggers inflammatory responses by the innate immune system. Release of HMGB1 by splenic MΦs occurs upon extensive splenic cell apoptosis, a feature commonly observed during sepsis. Indeed, HMGB1 is released into the extracellular milieu during sepsis and neutralization of this protein by monoclonal antibody treatment blocks sepsis development (19). Abundant splenic cell apoptosis is also typical in rodent malaria, at the peak of acute infection (20). In human malaria, endogenous HMGB1 serum levels are significantly higher in patients with severe disease compared to uncomplicated cases (21), suggesting that HMGB1 might also be involved in the development of immunopathology. Thus, it would not be surprising if acute immune response to *Plasmodium* and consequent immunopathology could be suppressed in great extent with neutralization of HMGB1.

Splenic MΦ receptors also include C-type lectin receptors (CLRs), such as dectin-1, mannose receptor, and dendritic cell-specific intercellular adhesion molecule-3-grabbing non-integrin (DC-SIGN). CLRs have multiple functions in the immune system, including pathogen recognition and neutralization (22). Additionally, the liver synthesizes mannose-binding protein (MBP) during infectious diseases. This protein activates the complement system in order to form the membrane attack complex (MAC), and, more importantly in the spleen, to opsonize microorganisms such as virus (23) or protozoan parasites such as *Trypanosoma cruzi* (24). Scavenger receptors (SRs), such as SR-A1 and MARCO, are also expressed in splenic MΦs and likewise bind both self and pathogen molecules – more specificities of these receptors will be discussed later in this review. Among class B SRs, CD36 is known to mediate the uptake of oxidized low-density lipoprotein (oxLDL) and apoptotic cells, but also promotes phagocytosis of *S. aureus* bacteria by peritoneal MΦs (25). However, CD36 mediates cytoadherence of *Plasmodium*-infected red blood cells (iRBCs) to microvascular endothelium (26), a process supposed to avoid parasite clearance inside the spleen. The role of CD36 in recognizing this parasite by splenic MΦs still needs to be fully elucidated. Of note, RpMΦs express constitutively this molecule, which implies a possible role for this receptor in antiplasmodial immunity. This is a clear example of a receptor capable of mediating the recognition of both self and non-self molecules, implicating RpMΦs with both blood homeostasis and control of blood-borne infections.

Among cytoplasmic PRRs, splenic MΦs express molecules from the NOD-like receptor (NLR) family (27). For example, disturbance of cellular ionic gradient activates the pyrin subfamily member NLRP3, leading to inflammasome complex formation and in consequence to the release of inflammatory cytokines IL-1β and IL-18. Hemozoin, a disposal product formed from hemoglobin digestion by *Plasmodium* parasites, triggers the NLRP3 inflammasome in bone marrow-derived macrophages (BMDMs) (28), mediating the production of proinflammatory cytokines by these cells. Furthermore, the NLRP3 inflammasome is activated in mouse RpMΦs and human peripheral monocytes during acute malaria – although large amounts of IL-1β are only produced after stimulation with LPS (29). Interestingly, in mice, this process is mediated by the purinergic P2 × 7 receptor which recognizes extracellular ATP. ATP accumulates in *Plasmodium*-iRBCs and is released into the extracellular milieu through ion channels in the erythrocyte membrane or upon iRBC rupture (30).

## Role of RpMΦs in Blood-Borne Infections

As stated previously, RpMΦs form a vast network inside the Rp, and although there is no consensus about the origin of RpMΦs, recent data indicate that these MΦs are maintained by local proliferation during physiological conditions (31). Conversely, in some pathological conditions, circulating monocytes are able to differentiate into RpMΦs (32). RpMΦ population comprises macrophage colony-stimulating factor (M-CSF)-dependent and M-CSF-independent cells (33). M-CSF-dependent RpMΦs are efficient phagocytes and produce proinflammatory cytokines such as TNF-α and type I IFNs and are highly responsive to prostaglandin E2 (PGE2). In contrast, M-CSF-independent BMDMs are less efficient phagocytes that produce high amounts of PGE2 (34). If this is a general feature of M-CSF-independent MΦ populations, M-CSF-independent RpMΦs might influence the activity of M-CSF-dependent RpMΦs.

Venous cords and sinuses render the splenic Rp bloodstream in a slow pace. This characteristic allows for the filtering function of the spleen and favors elimination of aberrant red blood cells (RBCs) or *Plasmodium*-iRBCs (35). Of note, development of RpMΦs relies on the expression of the transcription factor Spi-C, which is induced by free heme from RBC degradation (32). Thus, iron homeostasis – which conversely is controlled by RpMΦs – might play a role in RpMΦ development. Splenic structure also facilitates the control of numerous blood-borne infections by RpMΦs. For example, RpMΦs can recognize the capsular polysaccharide glucuronoxylomannan (GXM) from *Cryptococcus neoformans* and subsequently phagocytize the bacteria (36). RpMΦs can also eliminate *Streptococcus pneumoniae* under conditions of splenomegaly (37). However, these MΦs are permissive to intracellular growth of *Salmonella typhimurium* (38).

Red pulp macrophages have also been implicated in the control of blood-stage malaria (35). Nevertheless, in experimental *Plasmodium yoelii* infection, spleen remodeling facilitates iRBC adherence to the vascular endothelium and, in consequence, allows parasites to escape from phagocytes (15). Interestingly, a proportion of Rp phagocytes exhibit strong labeling for F4/80 and CD11c, a phenotype shared by RpMΦs and DCs (39). This population participates in the early clearance of *Plasmodium chabaudi* parasites, but it sharply declines at the parasitemia peak. RpMΦs have a slow turnover rate and possibly undergo cell death after ingesting *Plasmodium-*iRBCs due to the toxic effects of hemozoin. RpMΦs, which are primarily required to maintain tissue homeostasis, might be substituted by inflammatory phagocytes as well as by MΦs derived from inflammatory monocytes. An alternative explanation is downregulation of the F4/80 molecule upon MΦ activation, as reported during mycobacterial infection (40).

Several mechanisms mediate RBC recognition and clearance by RpMΦs. One of the most studied mechanisms is the antibody binding to altered self components such as Band 3 clusters (41) or phosphatidylserine residues exposed in the outer leaflet of RBC membrane (42). In these cases, natural antibodies and complement system proteins opsonize RBCs though recognition of Band 3 clusters or phosphatidylserine residues. Another important interaction involved in RBC phagocytosis by RpMΦs is the ligation of CD47 to Signal Regulatory Protein alpha (SIRPα) (43). CD47 is a self-molecule important to avoid clearance by phagocytes, which is ubiquitously expressed on many cell types, including RBCs. CD47 expression on RBCs is an inhibitory signal for phagocytosis (44), but RBCs expressing a modified isoform of this molecule are phagocytized by RpMΦs through SIRPα binding (43). Interestingly, the conformation-dependent anti-CD47 antibody 2D3 binds sickle RBCs preferentially (45), which might explain the enhanced phagocytosis of sickle RBCs inside spleen. A recent study showed that *P. yoelii* parasites preferentially infect young RBCs expressing high levels of CD47 and, in consequence, escape from splenic clearance (46). Furthermore, enhanced resistance to *P. yoelii* observed in CD47-deficient mice is associated with a larger population of RpMΦs that ingest more iRBCs than wild-type counterparts. These findings explain why individuals with mild genetic RBC disorders (e.g., sickle cell trait and glucose-6-phosphate dehydrogenase deficiency) are protected from lethal malaria due to enhanced RBC phagocytosis.

**Figure 2:**
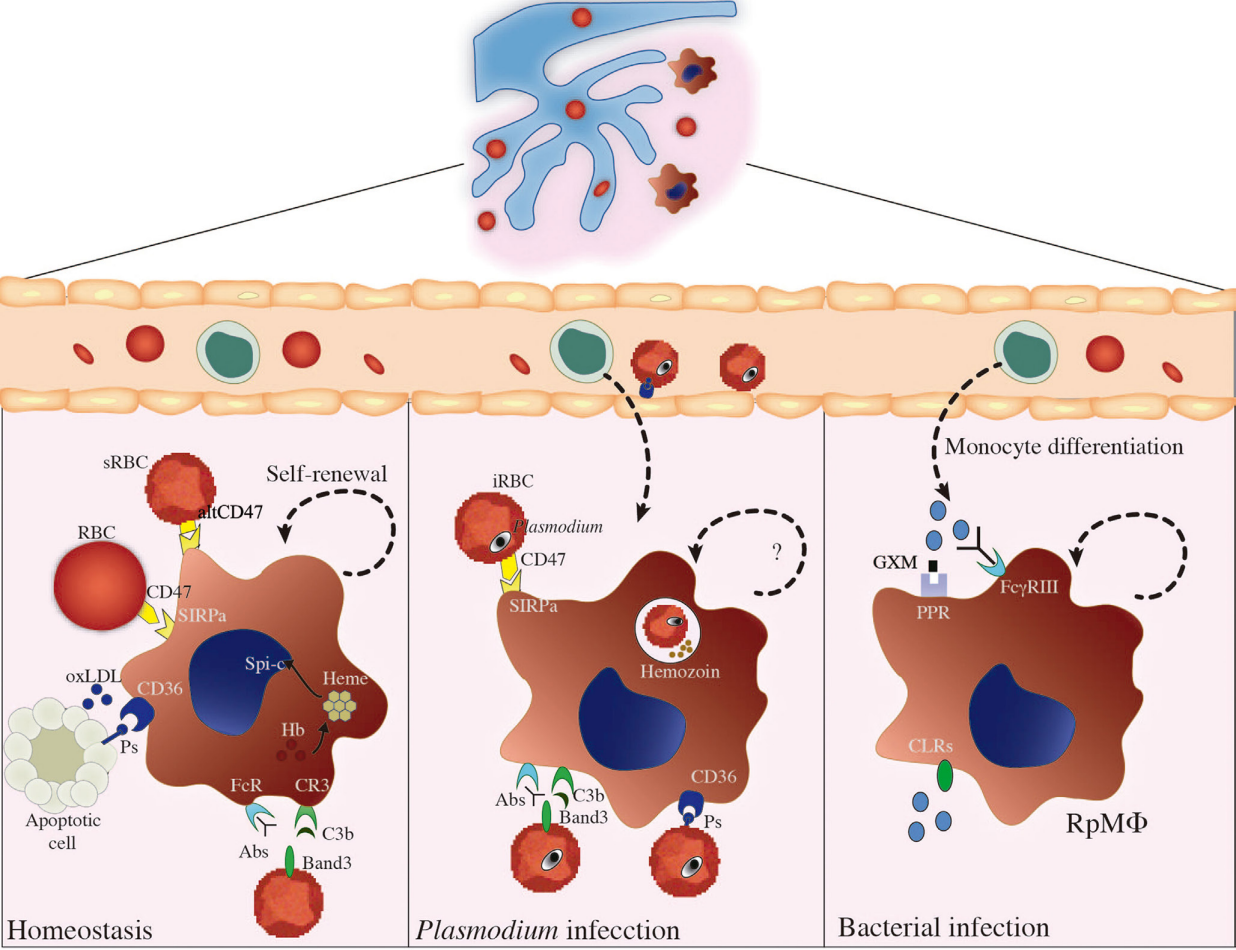
**RpMΦ biology during homeostasis and infection**. This figure summarizes the different roles of RpMΦs in maintenance of host homeostasis and in the control of different infections. In the absence of infection (left), RpMΦs play important roles in the uptake of apoptotic cells, oxidized LDL (oxLDL), or senescent RBCs (sRBCs) from the circulations, through interaction with receptors such as SIRPα, CD36, CR3, or FcRs. CD47 expression on RBCs is an inhibitory signal for phagocytosis mediated by SIRPα, but sRBCs expressing a modified isoform of this molecule (altCD47) are phagocytized by RpMΦs. CD36 binds to phosphatidylserine (PS) and, alternatively, to oxLDL. RpMΦs are also important for iron homeostasis, and conversely, iron homeostasis seems to control RpMΦ development, through the action of free heme on Spi-C transcriptional factor. In these situations, RpMΦs have the ability of self-renewal by proliferation. Beyond the task of maintaining blood homeostasis, RpMΦs contribute to control blood-borne infections such as malaria (center) or bacterial infections (right) lead to changes in RpMΦ function. *Plasmodium*-infected RBCs (iRBCs) are recognized through the same receptors that recognize sRBCs, such as SIRPα, CR3, FcRs, or CD36, inferring a role for RpMΦs in parasite clearance. However, the adherence of iRBCs to microvascular endothelium through CD36 prevents iRBC clearance inside the spleen. Interestingly, *P. yoelii* parasites preferentially infect young RBCs expressing high levels of CD47 and, in consequence, escape from splenic clearance. RpMΦs also present with other receptors such as CLRs and PPRs, which in conjunct with FcγRIII contribute to recognition and elimination of bacteria from circulation. RpMΦs can recognize the capsular polysaccharide glucuronoxylomannan (GXM) from *Cryptococcus neoformans* and subsequently phagocytize the bacteria. The ability of RpMΦ renewal during infections, however, is poorly understood, and substitution of dead RpMΦs for monocyte-derived RpMΦs is presumable.

Apart from being phagocytized by splenic MΦs, *Plasmodium*-iRBCs are also destroyed intravascularly as a consequence of plasma membrane damage upon release of free merozoites. Hemozoin, a disposal product formed from hemoglobin digestion by parasites, is released from lysed iRBCs. Furthermore, a massive destruction of non-infected RBCs occurs during blood-stage malaria, leading to increased hemoglobin levels in circulation [reviewed in Ref. (47)]. Another example of hemolysis induced by infections is observed in septicemia caused by *Escherichia coli*, which produces exotoxin α-hemolysin (Hlyα) (48). Evidencing RpMΦs crucial role in neutralizing toxic effects of hemoglobin, these MΦs have high levels of intracellular heme due to RBC phagocytosis (2) and of free hemoglobin through the scavenger receptor CD163 (49). The enzyme heme-oxygenase 1 (HO-1) plays an important role in degrading free heme, which in excess causes toxicity to MΦs (50). Importantly, RpMΦs are able to control pathogen burden through control of iron availability. For example, RpMΦs express the natural resistance associated macrophage protein-1 (NRAMP1) that is associated with protection against intraphagosomal pathogens, such as *Mycobacterium bovis* BCG, *Leishmania donovani*, or *S. typhimurium*. This molecule is a pH-dependent metal transporter localized in phagosomal compartments, which reduces intraphagosomal iron levels derived from RBC phagocytosis (51). NRAMP1 synthesis is upregulated in IFN-γ-activated MΦs (52), a condition likely to occur during acute blood-borne infections. RpMΦs also limit pathogen iron uptake through TLR-mediated release of lipocalin-2, which can form complexes with pathogen-secreted siderophores – molecules that help the collection of iron available for pathogens (53). RpMΦs involvement in controlling excessive immune responses is suggested by studies on autoimmune syndromes, while a similar participation in infectious diseases remains to be established. For instance, RpMΦs constitutively express peroxisome proliferator-activated receptor-γ (PPAR-γ), which might be important to curb excessive immune responses to pathogens, in a similar manner to PPAR-γ expressed on lung MΦs upon *S. pneumoniae* infection (54). RPMΦs can also prevent autoimmunity by producing anti-inflammatory cytokines such as TGF-β and IL-10 and by inducing generation of regulatory T (Treg) cells (55). Of note, there are many T cells scattered in Rp (55), and this population participates in acute immune responses to infections, such as blood-stage malaria (39). We present an illustrated scheme of the different roles of RpMΦs in homeostasis and disease in Figure 2.

## MZMΦs and MMMΦs Role in Blood-Borne Infections

Marginal zone macrophages and MMMΦs have unique characteristics that contribute to rapid phagocytosis of pathogens and other particles. Thus, these MΦs act like scavenger cells, developing pro- or anti-inflammatory responses depending on the nature of the interaction. MZMΦs express SIGNR1 that binds to yeasts and the yeast-derived particle zymosan (4), to bacteria such as *Mycobacterium tuberculosis* (56), *S. pneumoniae* (57), *E. coli*, and *S. typhimurium* (58), and to virus such as human immunodeficiency virus (HIV) (4). This receptor recognizes carbohydrate antigens from blood-borne pathogens and mediates their subsequent internalization into phagosomes (4). Although SIGNR1 in peritoneal MΦs cooperate with dectin-1 in zymosan uptake (59), these innate receptors colocalize poorly in MZMΦs (60). Similar to classical complement pathway activation, but independently of antibodies, SIGNR1 also binds C1q and assembles the complex C4bC2a or C3 convertase that catalyzes C3b opsonin formation (61). This mechanism was shown to provide resistance to intravenous *S. pneumoniae* infection.

Expression of the scavenger receptor MARCO is upregulated in different MΦ populations, especially in MZMΦs and MΦs in the medullary cord of lymph nodes (3). MARCO was firstly reported to bind and mediate uptake of Gram-negative bacteria and also to recognize oxLDL [reviewed in Ref. (62)]. The structure of MARCO is similar to that of the Scavenger Receptor A1 (SR-A1, CD204), which plays a role in bacteria and virus recognition (3). TLR-mediated activation of BMDMs stimulates MARCO-mediated phagocytic activity (63). Furthermore, MARCO in MZMΦs directly binds and mediates phagocytosis of *E. coli* and *S. aureus* bacteria (3). TLR engagement leads to activation of transcriptional mechanisms that increase phagocytosis and cell activation, and MARCO seems to work in conjunct with TLRs in order to mediate pathogen control (64).

**Figure 3:**
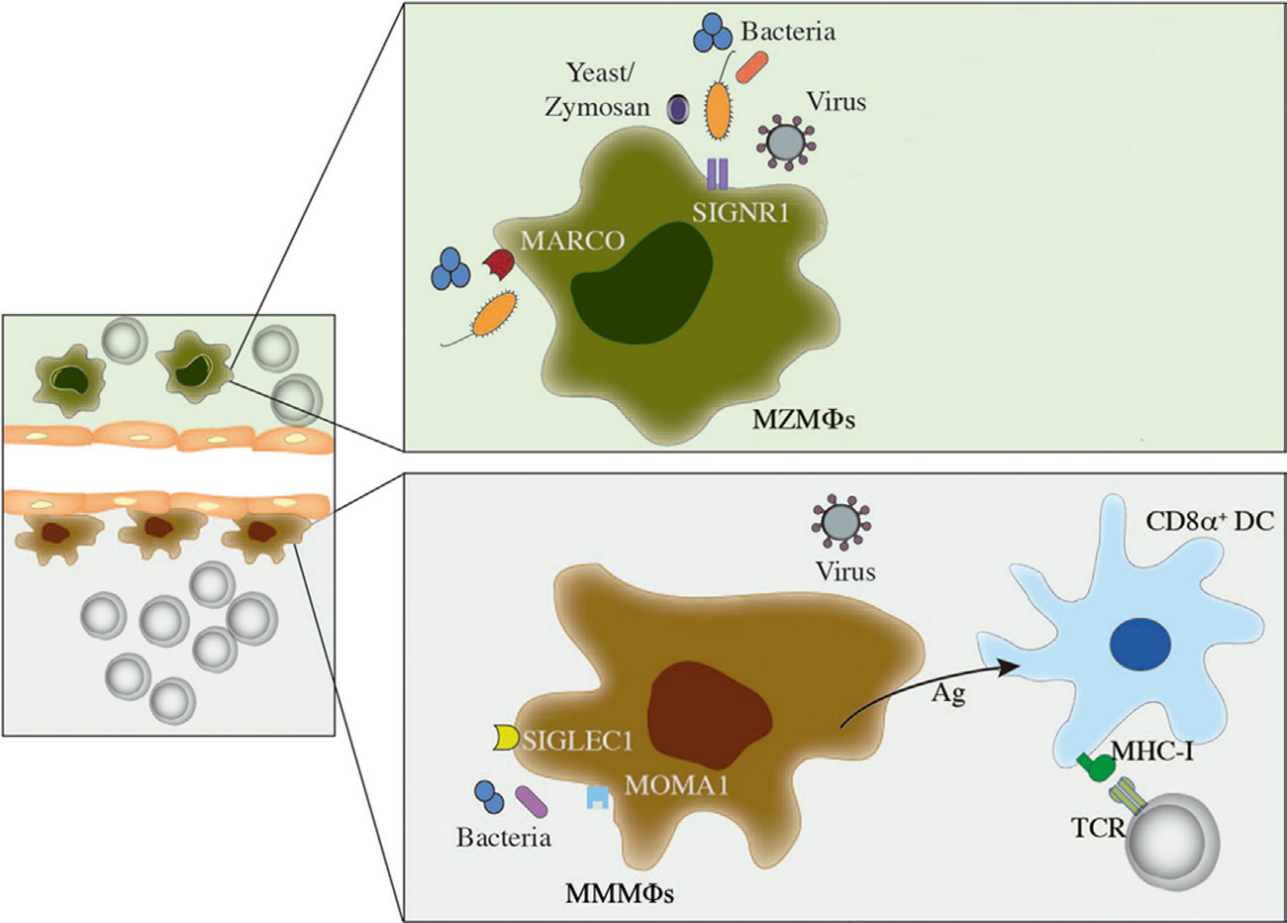
**Role of MZMΦs and MMMΦs during infection**. In this figure, a brief description on how MZMΦs and MMMΦs are able to recognize and mediate protection against blood-borne pathogens is shown. MZMΦs (above) can recognize bacterial and viral infections by receptors such as MARCO or SIGNR1, which usually induce internalization and further pathogen degradation. A similar feature can be depicted for MMMΦs, where MOMA-1 or SIGLEC can mediate pathogen recognition and elimination from circulation. MMMΦs can also interact with CD8α^+^ dendritic cells (DCs), which ultimately lead to CD8^+^ T-cell activation.

Marginal zone macrophages and MMMΦs are fundamental in the early control of *Listeria monocytogenes* bacteremia, as evaluated by depletion of these MΦs using a low dose of clodronate liposomes (65). T-cell responses are not affected in this experimental model, ruling out the participation of MZMΦs and MMMΦs as antigen-presenting cells. Similar findings were reported during infection with *Neisseria meningitidis* (64), thus it is likely that these MΦs have a direct role in the elimination of bacteria from circulation. Conversely, adenoviruses colocalize with MZMΦs as soon as a few minutes after intravenous injection in mice (66). MZMΦs and MMMΦs play a similar role in lymphocytic choriomeningitis virus (LCMV) infection, corroborating the importance of these MΦs in first-line antiviral defense (67). On the other hand, localization of MZMΦs and MMMΦs in the interface between the bloodstream and lymphocyte-rich zones makes them suitable to bridge innate and adaptive immunity in several situations. For instance, mice lacking SRs MARCO and SR-A1 show a defective microarchitecture of the splenic MZ and an impaired T-independent type 2 response when challenged with pneumococcal polysaccharide (68). MMMΦs also collaborate in cytotoxic T-cell activation by transferring antigen directly to CD8α^+^ DCs, which are specialized in cross-presentation to CD8^+^ T cells (69). This observation supports the use of the MMMΦs antigen-concentrating capacity in therapeutic strategies for the development of antitumor immunity. The different roles of MZMΦs and MMMΦs in blood-borne infections are shown in Figure 3.

## Reciprocal Influence of Splenic Microenvironment and MΦs

In several aspects, splenic MΦs shape splenic structure and/or microenvironment. The development of splenomegaly is typical in blood-borne infections, and it is characterized by profound changes in splenic microarchitecture, including remodeling of Rp (1). Given this, splenic MΦs are expected to play a prominent role in the recruitment of different cell types during acute immune responses. For example, RpMΦs recruit neutrophils into the spleen during early *Candida* infection by releasing CXCL1 and CXCL2, through autophagy-mediated depletion of the NF-κB inhibitor molecule A20 (70). Another example is the arresting of T cells inside the Rp during acute *Plasmodium* infection (39). RpMΦs may produce CXCR3- and/or CCR5-binding chemokines by a mechanism similar to that observed during early *Candida* infection – CXCR3 and CCR5 are the main upregulated chemokine receptors in splenic CD4^+^ T cells during acute blood-stage malaria (71). However, splenic MΦs might also act on splenic microenvironment after an acute infection. For example, apoptotic cell uptake induces CCL22 production by MMMΦs, which in turn induces Foxp3^+^ Tregs and DCs recruitment and accumulation, leading to a state of tolerance (72). Since the accumulation of apoptotic cells is a normal feature after acute blood-borne infections (20), a similar mechanism possibly takes place. RPMΦs can also prevent autoimmunity by producing anti-inflammatory cytokines such as TGF-β and IL-10 and by inducing the generation of Treg cells (55). These cytokines may be important – besides limiting autoimmunity – to curb an excessive immune response that could be dangerous to the host after pathogen clearance.

Conversely, the splenic structure and its microenvironment seem to play pivotal roles in MΦ homing and function. For instance, arrangement of sinusoidal endothelial cells inside Rp hampers the circulation of aging and/or iRBCs (1), facilitating their trapping inside Rp and posterior phagocytosis by RpMΦs. Importantly, the cytokine milieu in the microenvironment – which varies throughout an acute infection – may also dictate RpMΦ function. Classic M1 MΦs have an enhanced capacity to accumulate iron, which positively influences the maintenance of these cells in a proinflammatory state. On the other hand, alternative M2 MΦs have an increased ability to release iron, and increased iron availability in the microenvironment seemingly favors tissue remodeling [reviewed in Ref. (73)]. These effects can easily be associated with RpMΦs especially considering their role in iron uptake (1), thus it is possible that RpMΦs play distinct roles as M1- or M2-skewing microenvironments may occur during the beginning of an acute blood-borne infection or after pathogen clearance, respectively. Furthermore, the MZ contains a large number of resident cells that apparently depend on each other for their localization, thereby establishing and maintaining MZ integrity (1). For example, studies in which B cells were absent from the time of birth or in which they are depleted led to disappearance of MZMΦs and MMMΦs (74). Thus, the continuous interaction between resident and transmigrating cells inside the spleen MZ is crucial for efficient homing of MZMΦs and MMMΦs as well as for efficient removal and destruction of blood-borne pathogens by these cells. Lymphotoxin (LT) and TNF also influence the dynamics of MZMΦs and MMMΦs. *L. donovani*-infected mice have profound changes in the splenic MZ including loss of MZMΦs, which depend on TNF signaling that may increase MZMΦs susceptibility to parasite-induced cell death (75). These changes block lymphocyte traffic in the white pulp, impairing the development of an appropriate adaptive immune response. In another case, MZ B cells secrete LT-α1β2, and this leads to induction of a range of chemokines that could, in turn, influence lodging and retention of MZMΦs (76).

## Concluding Remarks

As discussed above, splenic MΦs (RpMΦs, MZMΦs, and MMMΦs) play important roles in the control of blood-borne infections and shape several aspects of innate and adaptive immune responses (Table 1). Thus, a clear concept on the nature of splenic MΦ populations can be drawn, in which their interplay with the splenic microenvironment guarantees efficient control of blood-borne pathogens and maintenance of homeostasis following these infections. At the same time, the splenic structure is likely fundamental for proper localization and function of these MΦs. However, several questions on the nature and function of these cells are still unanswered, especially on (a) the development of splenic MΦs during embryogenesis, (b) the exact signals required for the homeostatic maintenance of these cells, and (c) the extent of how important each of these subsets are for the development of immunity against blood-borne infections. The development of mouse models to accurately study the distinct roles of RpMΦs, MZMΦs, and MMMΦs as well as the development of more detailed studies on signaling pathways and epigenetic modifications on genes involved in the function of these cells will be of great utility to solve these questions.

**Table 1:** **Overview of splenic MΦ subsets**.

MΦ type associated markers	Connection to immune response	Associated pathogens
**RpMΦs**		
F4/80^+/++^ (2), CD11b^low^ (2), CD68^+^ (2), and SIRPα^+^ (43)	• Uptake of aging or apoptotic RBCs (2) • Limitation of autoimmunity (IL-10 and TGFβ in resolution of inflammation) (55) • Induction of Tregs by IL-10 production (55) • Phagocytosis of blood-borne pathogens (35–38) • Iron homeostasis (1, 50–53)	*Plasmodium* (35)*, Cryptococcus neoformans* (36), *Streptococcus pneumoniae* (37), *Salmonella typhimurium* (38)
**MZMΦs**		
SIGNR1^+^ (3, 4), F4/80^+/−^ (3, 4), MARCO^+^ (3, 4), lymphotoxin, and TNF receptors (75, 76)	• Clearance of modified LDL (1) • TI-2 B cell responses (68) • Phagocytosis of blood-borne pathogens (3, 4, 65, 67)	*Staphylococcus aureus* (3), *Listeria monocytogenes* (65), *Escherichia coli* (3), HIV (4), LCMV (67)
**MMMΦs**		
SigLec-1^+^ (CD169^+^) (5), MOMA-1^+^ (5), F4/80^+/−^ (5), lymphotoxin, and TNF receptors (75, 76)	• Indirect activation of CD8^+^ T cells (69) • Phagocytosis of blood-borne pathogens (67)	*Listeria monocytogenes* (65), LCMV (67)

*A subdivision of splenic MΦs, detailing RPMΦs, MZMΦs, and MMMΦs associated markers, their connection to the systemic immune response, and associated pathogens. The respective references from each feature are detailed inside the table*.

## Conflict of Interest Statement

The authors declare that the research was conducted in the absence of any commercial or financial relationships that could be construed as a potential conflict of interest.
